# Combination of the Herbs Radix Rehmanniae and Cornus Officinalis Mitigated Testicular Damage From Diabetes Mellitus by Enhancing Glycolysis via the AGEs/RAGE/HIF-1α Axis

**DOI:** 10.3389/fphar.2021.678300

**Published:** 2021-06-28

**Authors:** Yuping Chen, Jing Chen, Anmei Shu, Liping Liu, Qin Wu, Juansong Wu, Siyuan Song, Weiping Fan, Yihui Zhu, Huiqin Xu, Jihu Sun, Liucai Yang

**Affiliations:** ^1^ Department of Basic Medical Science, Jiangsu Vocational College of Medicine, Yancheng, China; ^2^ College of Pharmacy, Nanjing University of Chinese Medicine, Nanjing, China; ^3^ Hanlin College, Nanjing University of Chinese Medicine, Taizhou, China; ^4^ College of Pharmacy, Jiangsu Vocational College of Medicine, Yancheng, China; ^5^ College of Clinical Medicine, Jiangsu Vocational College of Medicine, Yancheng, China; ^6^ College of Science and Technology, Jiangsu Vocational College of Medicine, Yancheng, China

**Keywords:** radix rehmanniae, cornus officinalis, diabetic reproductive disturbance, glycolysis, AGEs, RAGE, HIF-1α

## Abstract

Radix Rehmanniae and Cornus Officinalis (RR-CO) have been widely used as “nourishing Yin and tonifying kidney” herb pairs for the treatment of diabetes mellitus (DM) and its complications in traditional Chinese medicine (TCM). Based on the theory of “kidney governing reproduction” in TCM, the aim of this study was to investigate the therapeutic effects of RR-CO on DM-induced reproduction damage through regulating testicular glycolysis. Moreover, the regulation of AGEs/RAGE/HIF-1α axis on the testicular glycolysis process has also been studied. Spontaneous DM model KK-Ay mice were used to investigate the protective effect of RR, CO, RR-CO on DM-induced reproductive disturbances. RR, CO, RR-CO improved DM-induced renal and testicular morphology damages. Moreover, the impaired spermatogenesis, germ cell apoptosis and motility in testis induced upon DM were also attenuated by RR, CO or RR-CO, accompanied by an increased level of glycolysis metabolomics such as l-lactate, d-Fructose 1,6-bisphosphate, etc. Meanwhile, glucose membrane transporters (GLUT1, GLUT3), monocarboxylate transporter 4 (MCT4) expression, lactate dehydrogenase (LDH) activity, HIF-1α were upregulated by RR, CO and RR-CO treatment compared with the model group, whereas AGE level and RAGE expression were decreased with the drug administration. The RR-CO group was associated with superior protective effects in comparison to RR, CO use only. Aminoguanidine (Ami) and FPS-ZM1, the AGEs and RAGE inhibitors, were used as a tool drug to study the mechanism, showing different degrees of protection against DM-induced reproductive damage. This work preliminarily sheds light on the herb pair RR-CO exhibited favorable effects against DM-induced reproductive disturbances through enhancing testicular glycolysis, which might be mediated by AGEs/RAGE/HIF-1α axis.

## Introduction

With the rapid increase in the number of diabetic patients and moreover a younger age at onset, recent attention has been drawn to the impact of diabetes mellitus (DM) on reproduction damage (Jangir and Jain, 2014). About 90% of men with diabetes have sexual dysfunction, decreased libido, erectile dysfunction, and even infertility (Ding et al., 2015). It has been reported that a lower pregnancy rate is related to diabetes, and therefore diabetes is a critical factor that affects male fertility potential. Semen analysis of diabetic men showed decreased sperm motility and density, and abnormal morphology. Interestingly, compared with a healthy control group, even if the semen parameters of diabetic men are normal, there was still a higher degree of damage to nuclear and mitochondrial sperm DNA (Imani et al., 2021). Furthermore, the levels of testosterone and gonadotropins in diabetic men were decreased due to the effect of reduced insulin on the hypothalamic-pituitary-gonadal axis (Rocha et al., 2014).

**GRAPHICAL ABSTRACT:**
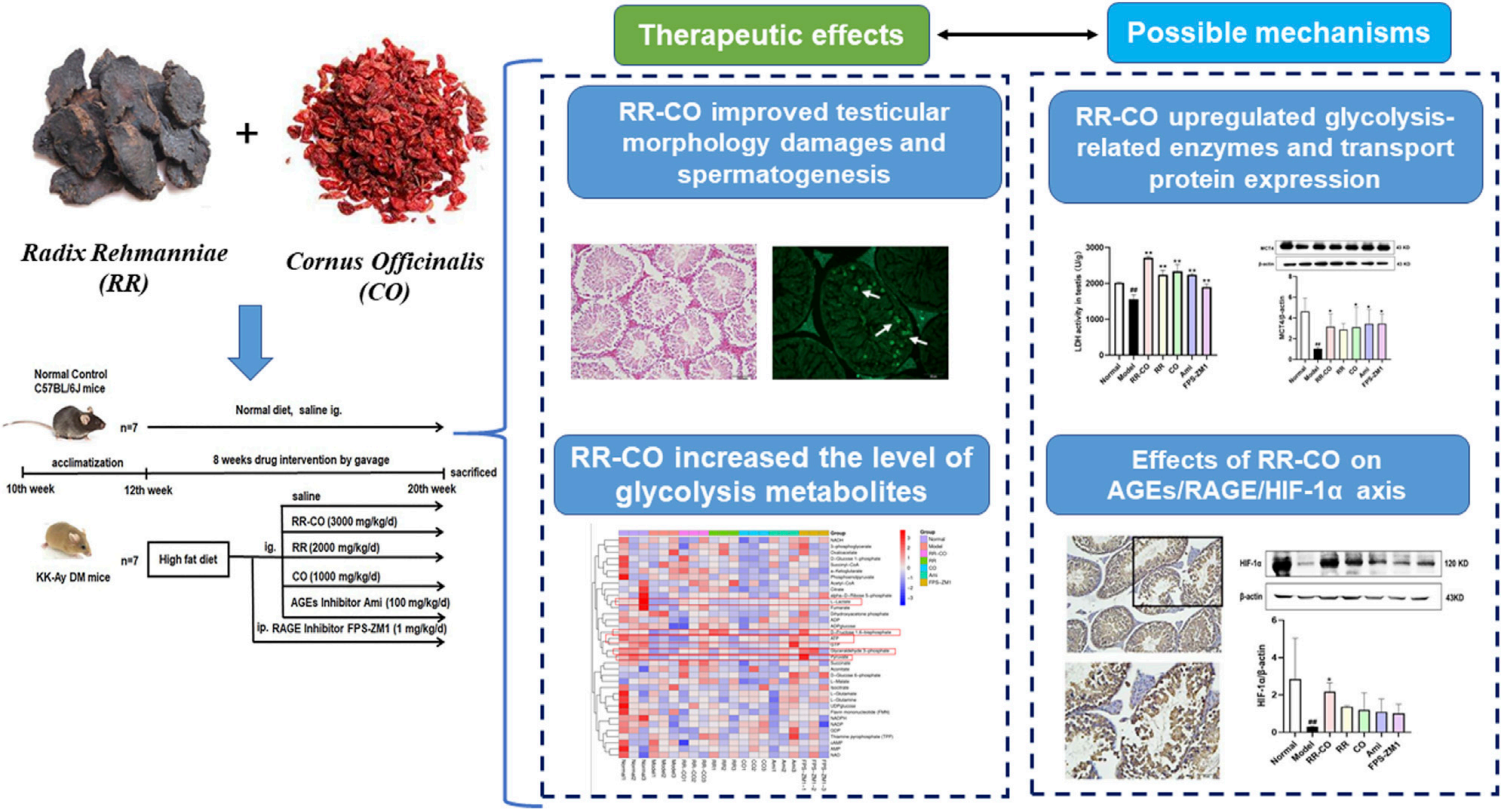


Recently, clinical and animal studies have shown that testicular metabolism is essential for spermatogenesis and maturation (Dias et al., 2014; Alves et al., 2015). Within the testis, Sertoli cells, (SCs) often termed “nurse” cells, are responsible for converting glucose to lactate, which is the preferred substrate for energy requirements for germ cell development. Metabolic cooperation between SCs and germ cells is important for spermatogenesis (Rato et al., 2012). The development and maturation of sperm are highly dependent on anaerobic glycolysis, and metabolic alterations from diabetes may be responsible for the disruption of spermatogenesis, contributing to male reproductive disorders.

Advanced glycation end products (AGEs) are irreversible post-translational modifications of proteins formed by non-enzymatic glycation reactions between glucose and proteins. Levels of AGEs are increased under conditions of long-term hyperglycemia, and they play a pathological role in diabetes and its complications (Singh et al., 2014). Testicular tissue is a hypoxic organ, and HIF-1α is a transcription factor that regulates cell glycolysis in a hypoxic environment. HIF-1α can increase glucose uptake and glycolysis by upregulating components of the glycolytic pathways, including the glucose transporter GLUT1, hexokinase (HK), lactate dehydrogenase (LDH), and the monocarboxylate transporter 4 (MCT4). Studies have shown that AGEs and their active precursor, methylglyoxal (MGO), can inhibit the formation of dimers of HIF-1α and HIF-1β by inhibiting the transcription cofactor (p300/CBP) (Catrina, 2014; Choudhry and Harris, 2018). This reduces binding to the hypoxia response element (HRE), thereby reduces transcriptional activity of HIF-1α for exposure to a diabetic environment. Furthermore, AGEs interact with their receptor (RAGE), which initiates downstream signaling pathways and increased reactive oxygen species (ROS) production. Prolyl hydroxylase is further activated by ROS, promoting HIF-1α degradation (Xiao et al., 2013). Hence, these studies suggest that AGEs, RAGE and HIF-1α comprise an important pathway of influencing glucose metabolism in testicular cells during diabetes.

Corni Fructus (Cornus officinalis Sieb. et Zucc., CO, family: Cornaceae), also known as “Shan Zhu Yu” in Chinese, is delivered as a tonic, with proposed functions of nourishing the liver and kidney, and has a history for the treatment of DM (Park et al., 2015; Bi et al., 2019). It is also used in TCM prescriptions such as Zan Yu dan or Liuwei Dihuang Pill to nourish Yin for the treatment of spermatorrhea and reproductive disorders (Huang et al., 2018). Rehmanniae Radix (*Rehmannia glutinosa* Libosch, RR, family: Scrophulariaceae), the root of Rehmannia glutinosa Libosch, known as “Shengdi” in Chinese, has reported health benefits that include clearing away heat, cooling of the blood, nourishing Yin and producing body fluid, and is often used to treat inflammation and metabolic diseases including high blood pressure and diabetes (Kim et al., 2017; Qi et al., 2019). The RR-CO herb pair is utilized for “Zi Yin Bu Shen,” for nourishing Yin and strengthening the kidney. It is often used in TCM formulations, such as Shen-qi-wan and Zi-cui-yin (Lv et al., 2016). According to TCM theory, the “Kidney Governs Reproduction” is a component of the basic understanding of the physiological and pathological features of human reproductive functionality (Huang et al., 2019). Hence, deficiency of kidney-yin is linked to core pathogenesis of male reproductive injury in diabetes (Lv., 1997).

Our previous studies have shown that administration of RR-CO can attenuate DM-induced renal and male reproductive damage in DM mice by inhibiting the AGEs-RAGE pathway (Chen et al., 2016). It has been reported that Shen-qi-wan, which contains RR-CO, can reduce renal fibrosis by upregulating HIF-1α and GLUT1 mRNA in the renal cortex (Oka et al., 2011). Thus, we hypothesized that RR-CO might regulate glycolysis of testicular cells to improve diabetes-induced reproductive damage by influencing the levels and activities of AGEs, RAGE, and HIF-1α. Herein, we investigated the protective effect of the herb combination, RR-CO, on diabetic reproductive damage based on the regulation of glycolysis. In addition, the role of AGEs-RAGE and the HIF-1α axis in glycolysis was also explored.

## Materials and Methods

### Reagents and Antibodies

**FIGURE 1:**
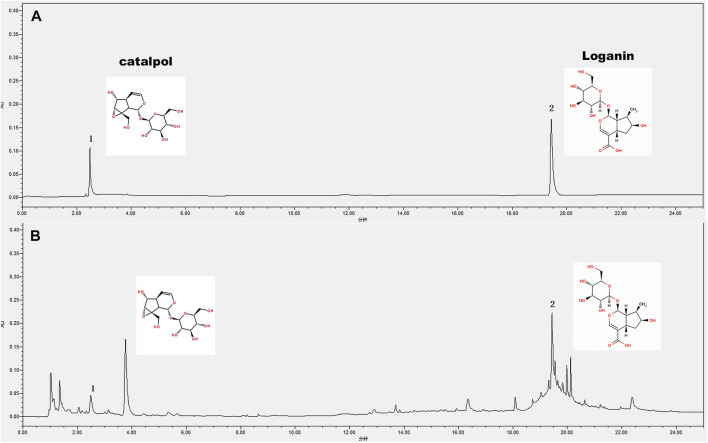
HPLC Charts for determination of main compounds in RR-CO including catalpol and loganin **(A)** The catalpol and loganin standard sample **(B)** The RR-CO decoction.

Raw herbs of RR and CO were purchased from Jiangsu Haichang Chinese Medicine (Jiangsu, China, Batch No. 190101) and identified by a Professor from Nanjing University of Chinese Medicine, the voucher number of CO (No.20170505) and RR (No.20181020) was deposited. The main effect components loganin and catalpol were determined by HPLC for quality control by referring to pharmacopoeia (Figure 1). The herbs were cut into small pieces and soaked in 10 volumes of distilled water for 30 min. They were then boiled under reflux for 1 h twice. For the RR-CO group, the two raw herbs were mixed at a clinically common concentration of a 2:1 ratio. The final concentration of RR, CO, RR-CO is 200 mg/ml, 100 mg/ml and 300 mg/ml respectively. Loganin (98% purity, Batch No. M-010–160,516) and Catapol (98% purity, Batch No. Z-005–160,502) standard samples were purchased from Chengdu Herbpurify Co., Ltd. Aminoguanidine (Ami) (98% purity, Batch No. 079K1734V) were obtained from Sigma. FPS-ZM1 (100% purity, Batch No.2908726) was purchased from Calbiochem (Shanghai, China). Antibodies against RAGE (Batch No. ab3611), GLUT1 (Batch No. ab162), and GLUT3 (Batch No. ab41525) were purchased from Abcam, and the HIF-1α antibody (Batch No. 36169) was purchased from Cell Signaling Technology. Antibodies against MCT4 (Batch No. sc-376465) were obtained from Santa Cruz Biotechnology. Antibodies against LDHC (Batch No. 19989-1-AP), PFK (Batch No. 13389-1-AP) were purchased from Wuhan Proteintech Group (Wuhan, China).

### Animal Model Establishments

**FIGURE 2:**
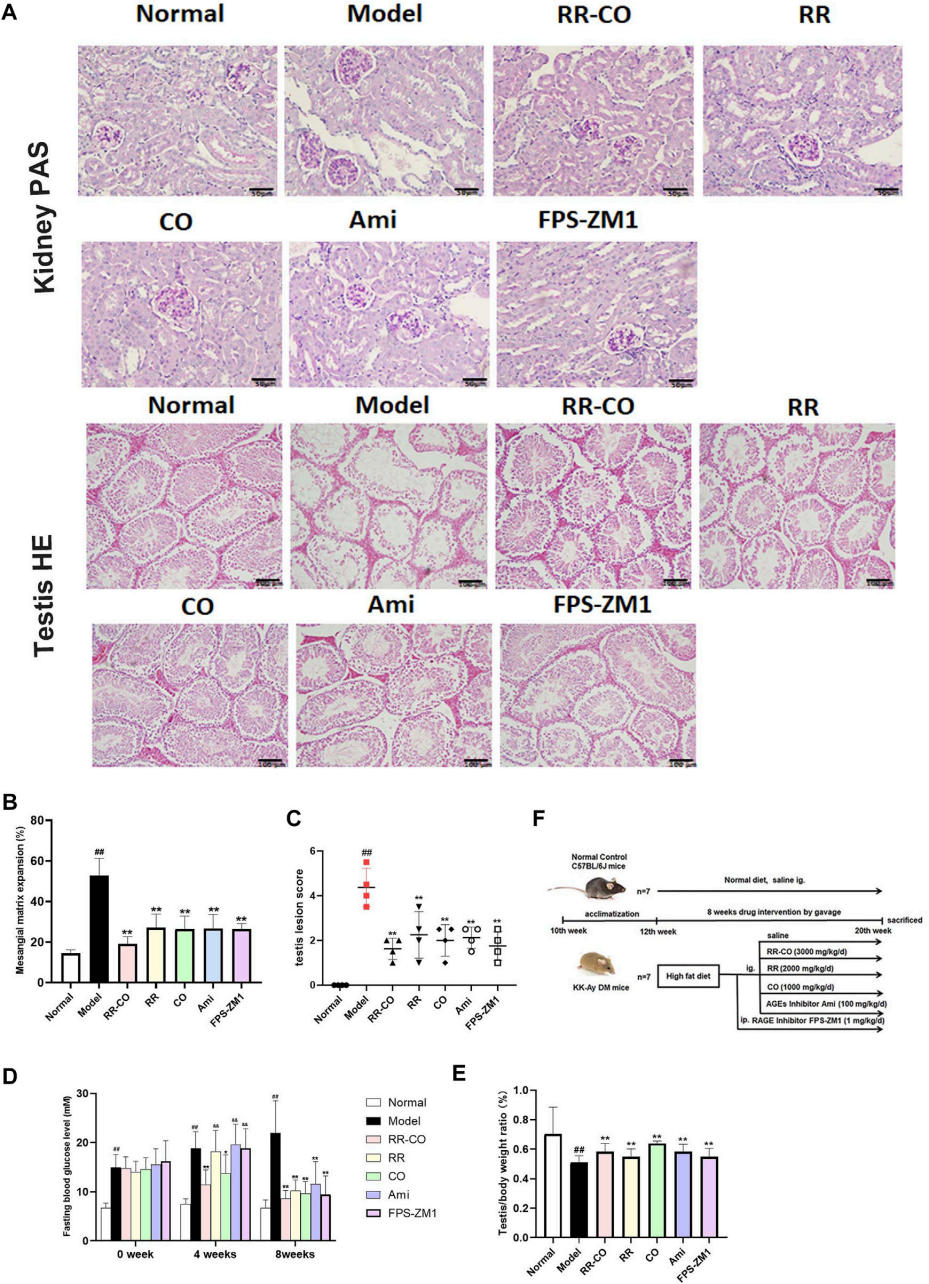
RR-CO improved DM-induced renal and testicular morphology damages **(A)** PAS staining of kidney and HE staining of testis (200× magnification) **(B)** The glomerular mesangial matrix expansion was measured according to the PAS staining **(C)** Statistical chart of testicular lesion score **(D)** Fasting blood glucose levels **(E)** The testis/body weight ratio **(F)** Flowchart of the animal study. Significance: ^##^ *p* < 0.01 vs. normal group; *p < 0.05, **p < 0.01 vs. model group. ^&&^ *p* < 0.01 vs. RR-CO group.

10-week-old male C57BL/6J and KK-Ay mice (License No. 2014–0004) were purchased from Beijing Huafukang Biotechnology Co., LTD. (Beijing, China). All mice were bred at (25 ± 1)°C and (55 ± 5) % relative humidity for 2 weeks before experiments. KK-Ay mice were fed a high-fat diet (425 kcal/100 g, containing 10% fat, containing 17.5% crude protein, 17.9% crude fat, 3.1% crude fiber, 4.5% crude ash, 8.5% moisture, 0.88% calcium, 0.58% total phosphorus and 48.5% Nitrogen-free extract), while C57BL/6J mice were fed a normal diet. All animal experiments are approved by the Animal Ethics Committee of Nanjing University of Chinese Medicine (Code: 201903A019) and kept in standardized laboratory animal centers. KK-Ay mice were randomly divided into the following groups (*n* = 7): the herb pair RR-CO group, RR group, CO group, inhibitor against AGEs (Ami) group and RAGE inhibitor (FPS-ZM1) group. All these groups were fed a high-fat diet. RR-CO group, RR group, CO group, Ami group was orally given the corresponding agent for 8 weeks. The intragastric doses were RR-CO (3000 mg/kg/d), RR (2000 mg/kg/d), CO (1000 mg/kg/d), Ami (100 mg/kg/d) respectively. FPS-ZM1 is given intraperitoneally at a dose of 1 mg/kg/d. Seven C57BL/6J mice fed a normal diet and administered with an equal volume of saline were set as a control group. Took blood sample from the tail vein of the mice, and then measured the fasting blood glucose (FBG) with a glucometer every 2 weeks. At the end of the experiment, the mice were sacrificed by cervical dislocation, the blood was collected from the orbit, and the blood was left for 30 min at room temperature, centrifuged, and the serum was collected. In addition, testicular tissue was retained, weighed, and the ratio of the bilateral testis to body weight was calculated. The flowchart of the animal study design was shown in Figure 2F.

To investigate the effect of RAGE on the testicular glycolysis, another 8-week-old male KK-Ay mice (License No. 2019–0008) were transfected with RAGE-expressing adenovirus (rAAV-mRAGE-IRES-ZsGreen) after 2 weeks of acclimatization (Animal Ethic No. 201910A031). rAAV-mRAGE-IRES-ZsGreen was constructed by KeyGEN Biotech Corp., Ltd. (Nanjing, China). The gene No. of mouse RAGE is NM_007425.3. Briefly, 200 μL of rAAV-mRAGE-IRES-ZsGreen (1×10^12^ vg/ml) were injected into KK-Ay mice through the tail vein every other day for 2 weeks to make RAGE overexpression. After that, the mice were divided into rAAV-RAGE (*n* = 6) and RR-CO group (*n* = 6) with RR-CO 3000 mg/kg/d intragastric administration for 8 weeks. Furthermore, C57BL/6J and KK-Ay mice that were not injected with the virus were treated with gastric saline as normal and model control respectively (*n* = 6).

### Determination of Live Sperm Rate

The live sperm rate was measured as previously reported (Chen Y. et al., 2020). The cauda epididymis was homogenized in PBS (pH 7.2) and incubated at 37°C for 5 min to release sperm. Then the suspension was mixed with an equal volume of 1% trypan blue and quantified under a light microscope in 200× magnification. Live sperm completely excluded the dye, while dead sperm accumulated it, showing a blue head.

### Histological Morphology, Immunofluorescence and Immunohistochemistry Assay

Kidney and testis samples were fixed in 4% paraformaldehyde solution and then embedded in paraffin. The paraffin blocks were cut into 5 μm thick sections and stained with hematoxylin and eosin (HE). Representative images of renal and testicular sections were taken and the expansion coefficient of the glomerular matrix was evaluated by measuring the glomerular area with Image J software, and the area of 10 glomeruli was calculated for each section. The testis pathological score was evaluated according to the arrangement and morphology of seminiferous tubules. For Immunofluorescence staining, the 5 μm thick paraffin sections of mice testis was deparaffinized and rehydrated in xylene and ethanol, followed by incubation with proteinase K working solution at 37°C for 20 min. After rinsing with PBS, the samples were incubated with primary antibody against RAGE at a dilution ratio of 1:200 under room temperature for 2 h and then with a secondary antibody with FITC fluorescence. The confocal images were acquired with a Zeiss LSM 900 Operation confocal microscope. Positive staining of IHC or intensity of immunofluorescence was quantified by Image Pro Plus software.

### Evaluation of Apoptosis in KK-Ay Mice Testis by TUNEL Staining

Testicular apoptosis was detected by One Step TUNEL Apoptosis Assay Kit (Beyotime Biotechnology, batch No. C1086, Nantong, China). The testicular tissue wax block was deparaffinized and dehydrated, incubated with proteinase K at 37°C for 20 min, rinsed with PBS 3 times, and then incubated with the TUNEL reaction mixture at 37°C for 1 h. The number of TUNEL positive cells in the testis seminiferous tubules was counted in a 400-fold visual field and the apoptosis rate was calculated.

### Targeted Liquid Chromatography-Tandem Mass Spectrometry (LC-MS/MS)Analysis Detects Glucose Metabolites

Targeted LC-MS/MS analysis was employed to examine 36 glucose metabolites in the testes of mice. Extractions from testis samples were taken, weighed, and grounded in liquid nitrogen. Each sample is supplemented with 1 ml of precooled methanol/acetonitrile/water with a 2:2:1 volume ratio and equal amounts of Succinate-d6 and Alanine-D4 as an internal reference. The protein was incubated at −20°C for 1 h and centrifuged at 14000 g at 4°C for 20 min. The supernatant was taken and performed on Nexera X2 LC-30AD Ultra high-performance liquid chromatography system (Shimadzu, Japan) and QTRAQ 5500 Mass Spectrometer (AB SCIEX, United States). MultiQuant software was used to extract a chromatographic peak area and retention time. Calibration was performed using energy metabolite standards.

### Quantitative Real-Time PCR, Lactate Dehydrogenase Activity Assay and Western Blotting

LDH activity was measured by a commercial assay kit (Nanjing Jiancheng Bioengineering Institute, Batch No. A020-2–2). Trizol was used to extract total RNA from testicular tissue (Shanghai Yeasen BioTechnologies co., Ltd. Batch No. 10606ES60, China). RNA was reverse transcribed into cDNA using a reverse transcription kit (Shanghai Yeasen BioTechnologies co., Ltd. Batch No. 11123ES60, China). The fluorescent probe SYBR (Shanghai Yeasen BioTechnologies. Batch No. 11203ES08, China) was added to amplify the target mRNA. The primers are designed for target genes and reference gene β-actin are shown in Supplementary Table S1. Samples were executed in triplicate in each assay. The target mRNA expression was calculated following the formula 2^−ΔΔCt^. The western blot experimental procedure is based on our previous paper description (Chen Y. et al., 2020). In brief, testes were homogenized in RIPA buffer containing protease inhibitor (Solarbio, China). The protein concentration was measured using a BCA assay kit (Pierce, United States). Protein (30–50 μg/well) was separated by SDS-polyacrylamide gel and transferred to a PVDF membrane (Millipore, United States) that was then blocked with 5% BSA in Tris-buffered saline containing 0.05% Tween 20. Target proteins were detected by primary antibodies and subsequently by horseradish peroxidase-conjugated secondary antibodies. Use a chemiluminescence kit (Millipore, United States) to visualize the protein bands, and quantify their intensity by Image J software. The experiment was replicated three times.

### Statistical Analysis

Data was expressed as mean ± standard deviation (SD). SPSS19.0 software was used for one-way ANOVA analysis of variance, and the LSD *t*-test was used to analyze differences between groups. *p* < 0.05 indicates a statistical difference.

## Results

### RR-CO Improved Diabetes Mellitus-Induced Renal and Testicular Morphology Damages

In the theory of TCM, reproduction is closely related to kidney function, and accordingly, we have evaluated both renal and testicular lesions. Using a model of DM, Periodic Acid Schiff (PAS) staining showed a significant increase of rose-red patches, as an indication of glycogen accumulation and glomerulus mesangial expansion, compared with normal mice controls. Within the testis, hematoxylin-eosin (HE) staining indicated a marked reduction in the number of sperm cells, accompanied by a disruption of the seminiferous tubules for the DM model group. By comparison, control mice displayed normal testicular structure and complete seminiferous tubules.

The administration of RR, CO, or for the combined RR-CO group, or the inhibitor group Ami and FPS-ZM1 against AGEs and RAGE, improved glomerular mesangial expansion and testis pathological damage to varying degrees (Figures 2A–C). Moreover, the FBG levels at 0, 4, 8 weeks after drug administration was measured, and the ratio of bilateral testes to body weight at the 8th week was calculated. The FBG level in the DM model mice was significantly elevated (1.7-fold, 2.5-fold, and 3.3-fold, respectively) compared with those of normal mice at 0, 4, and 8 weeks (*p* < 0.01). However, this was significantly reduced by RR-CO RR, CO, or the inhibitor Ami, or FPS-ZM1 administration at the 4th and 8th week time points. The hypoglycemic effect experienced by the RR-CO group was better than that of the RR, Ami, or FPS-ZM1 groups at the 4th week time point, and was significantly different (*p* < 0.01) (Figure 2D). Compared with the normal group, the testis/body weight ratio decreased significantly in the DM mice model, but this recovered, to some extent, in the RR-CO, RR, CO, or inhibitor treatment groups (Figure 2E).

### RR-CO Partially Restored the Impaired Spermatogenesis and Sperm Motility Loss and Was Accompanied by an Inhibition of Aerobic Glycolysis Within Testis in the Diabetes Mellitus Model

**FIGURE 3:**
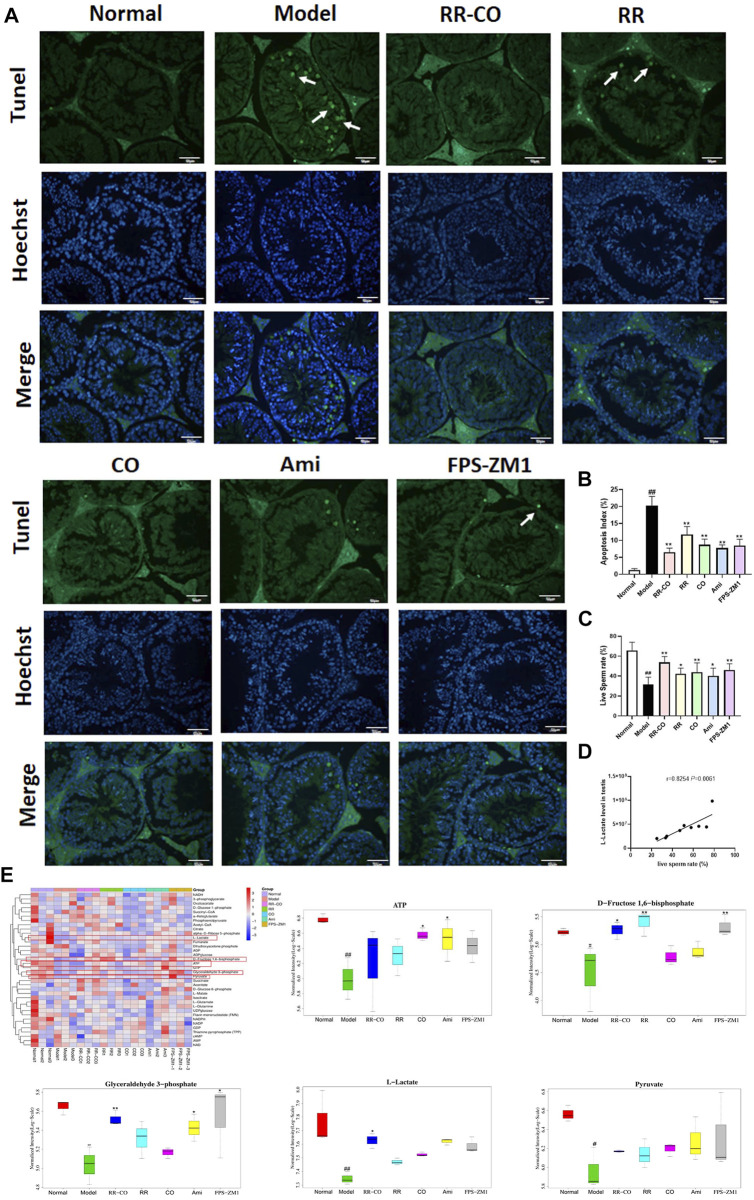
Effects of RR-CO on the testis apoptosis, glycolysis metabolites, and sperm motility **(A)** TUNEL assay was used to detect testicular apoptosis, of which apoptotic cells were indicated using white arrows, and the cell nuclei were subjected to Hoechst staining (200× magnification) **(B)** Apoptosis index for TUNEL assays **(C)** Live Sperm rate **(D)** Correlation analysis between l-lactate level and Live Sperm rate **(E)** Heat map of glycolysis metabolites measured using LC-MS/MS and statistical diagram of differential glycolysis metabolites. Significance: ^##^ *p* < 0.01 vs. normal group; *p < 0.05, **p < 0.01 vs. model group.

A TUNEL assay was used to detect the apoptotic status of seminiferous tubules in the testes. Levels of apoptotic cells within the seminiferous tubules of the control group were very few, while that of the DM model group were significantly increased. The apoptotic status was improved for each treatment group, with the most significant improvement with the RR-CO treatment group (Figures 3A,B). We screened 36 glucose metabolites using targeted LC-MS analysis. l-lactate, d-fructose 1,6-bisphosphate, ATP, glyceraldehyde 3-phosphate, and pyruvate were decreased in the testicular tissue of the DM model mice compared with the control group, indicating that glycolytic flux was inhibited in a diabetic environment. The level of glycolysis was improved to different degrees with each of the drug treatment groups, especially l-lactate, d-fructose 1,6-bisphosphate, and glyceraldehyde 3-phosphate levels which were increased in the RR-CO treatment group (Figure 3E) (*p* < 0.01, *p* < 0.05). Live sperm rate was used to assess testicular damage, and this was significantly decreased in the DM model group, but partly restored and increased with each of the treatment groups. There was a strong positive correlation between live sperm rate and l-lactate levels (Figures 3C,D) (*r* = 0.8254, *p* < 0.01).

### Effects of RR-CO on the Expression of Enzymes Related to Anaerobic Glycolysis and Glucose Transport

**FIGURE 4:**
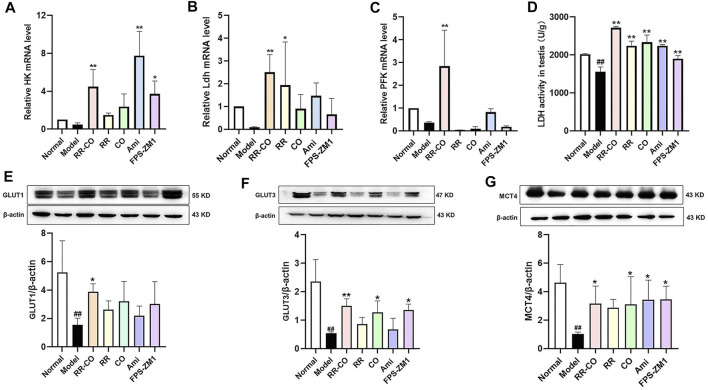
Effects of RR-CO on the expression of the aerobic glycolysis-related enzymes and transport protein **(A)** Relative HK mRNA level in testis **(B)** Relative LDH mRNA level in testis **(C)** Relative PFK mRNA level in testis **(D)** LDH activity in testis **(E)** The GLUT1 protein expression in testis **(F)** The GLUT3 protein expression in testis **(G)** The MCT4 protein expression in testis. Significance: ^##^ *p* < 0.01 vs. normal group; *p < 0.05, **p < 0.01 vs. model group.

Glycolytic flux is closely related to spermatogenesis, and is regulated by several rate-limiting enzymes and transporters. Hence, the expression of the most relevant glucose membrane transporters (GLUT1, GLUT3, and MCT4) and glycolytic rate-limiting enzymes, hexokinase (HK), LDH and phosphofructokinase (PFK) were evaluated within the testis. No significant differences were observed for testicular HK, LDH, or PFK mRNA expression levels between the control and DM group. However, testis LDH activity was drastically reduced, and this activity was recovered by RR-CO, RR, CO, or the inhibitor treatment (Figures 4A–D) (*p* < 0.01). There was also a significant reduction of GLUT1, GLUT3, and MCT4 expression in the DM model mice compared with the control group. The RR-CO treated group displayed increased expression of these three transporters, while for the FPS-ZM1 group, enhance GLUT3 and MCT4 expression was observed (Figures 4E–G) (*p* < 0.01).

### The Enhancing Effects of RR-CO on Testis Glycolysis Are Dependent on the AGEs/RAGE/HIF-1α Axis

**FIGURE 5:**
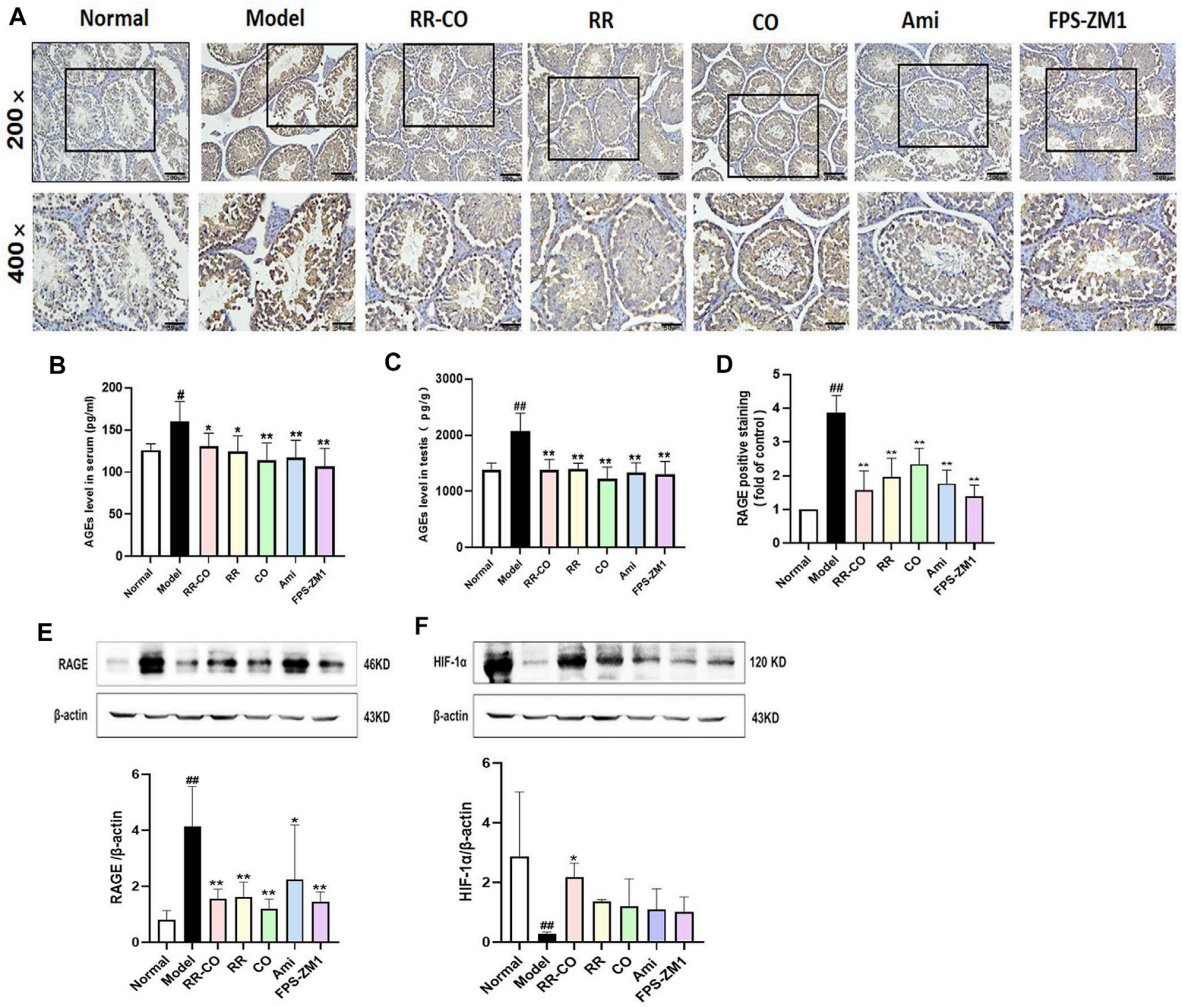
Effects of RR-CO on the AGEs/RAGE/HIF-1α axis **(A)** The expression of RAGE was detected by Immunohistochemistry (200× and 400× magnification) **(B)** AGEs level in serum detected by ELISA kit **(C)** AGEs level in testis detected by ELISA kit **(D)** Statistic Chart of RAGE positive staining of Immunohistochemistry analyzed by Image Pro Plus software **(E)** Western blot analysis of RAGE protein expression in the testis **(F)** Western blot analysis of HIF-1α protein expression in the testis. Significance: ^##^ *p* < 0.01 vs. normal group; *p < 0.05, **p < 0.01 vs. model group.

AGEs/RAGE is a key pathologic pathway that is activated with excessive blood glucose levels in DM and its complications. Since HIF-1α is closely related to the glycolysis involved in testicular spermatogenesis, we evaluated the levels of AGEs in serum and testis, and the protein expressions of RAGE and HIF-1α. Compared with the control group, the levels of AGEs in the serum and testis of the DM model group were higher, but administration of RR-CO, RR, CO, or the inhibitor Ami, FPS-ZM1 reduced AGEs deposition to varying degrees (Figures 5B,C) (*p* < 0.01, *p* < 0.05). Examination by Immunohistochemical and Western blotting techniques demonstrated that when compared with the control mice, the expression of RAGE increased in the DM model group (*p* < 0.01), but this increase was significantly attenuated in each of the treatment groups (Figures 5A,D,E) (*p* < 0.01). HIF-1α protein was also markedly decreased in the DM model mice compared with the control group, but RR-CO treatment was able to upregulate its expression (Figure 5F) (*p* < 0.01).

**FIGURE 6:**
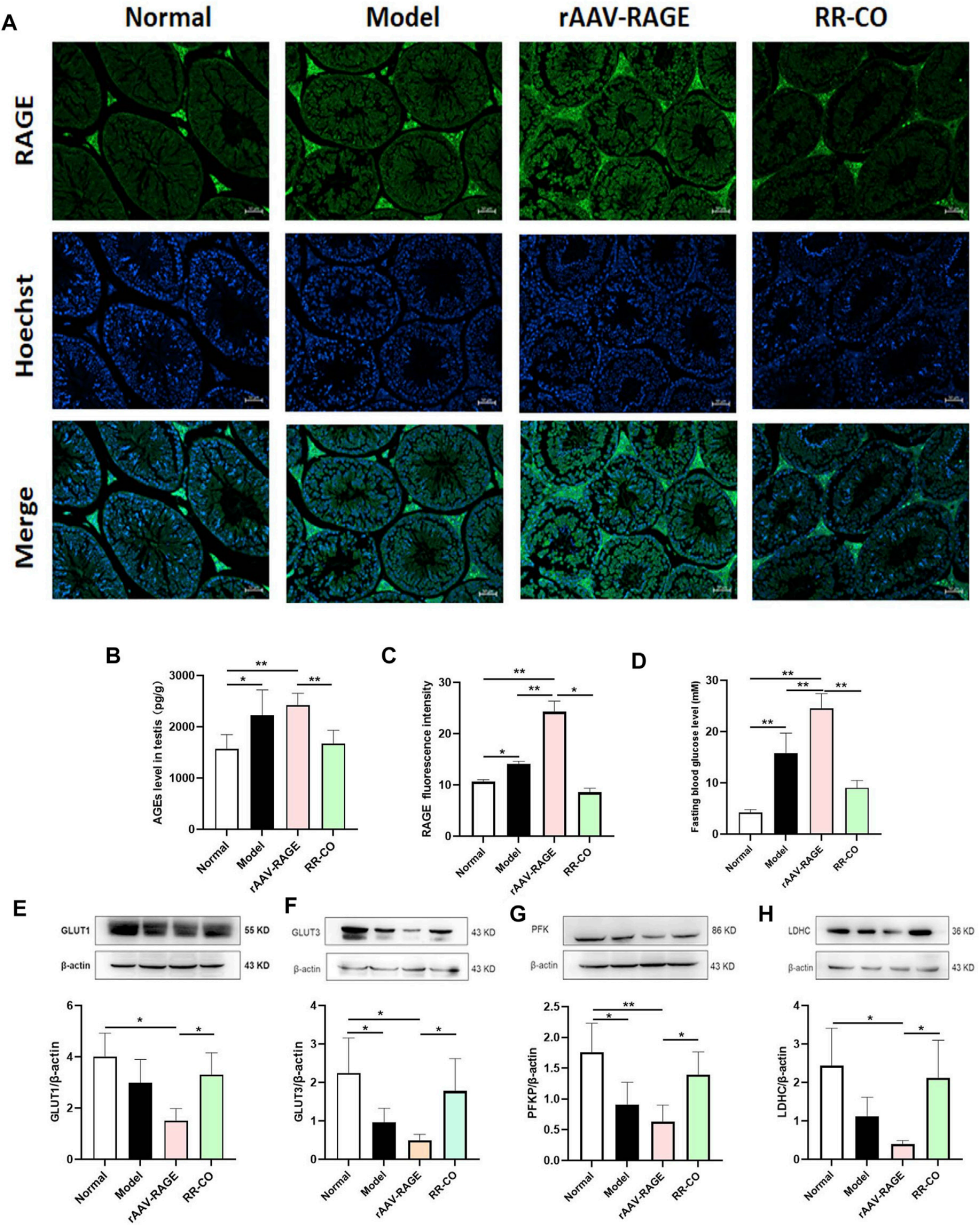
RAGE overexpression aggravated the testicular injury and glycolysis inhibition **(A)** The expression of RAGE was detected by Immunofluorescence **(B)** AGEs level in testis detected by ELISA kit **(C)** Statistical graph of RAGE immunofluorescence intensity **(D)** FBG level **(E)**Western blot analysis of GLUT1 protein expression in the testis **(F)** Western blot analysis of GLUT3 protein expression in the testis **(G)** Western blot analysis of PFK protein expression in the testis **(H)** Western blot analysis of LDHC protein expression in the testis. Significance: *p < 0.05, **p < 0.01.

To further verify the role of RAGE in testicular glycolysis, a model of RAGE overexpression in KK-Ay mice (rAAV-RAGE group) was established through adenovirus (rAAV-mRAGE-IRES-ZsGreen) tail vein injection. C57BL/6J and KK-Ay mice without the virus injection were set as normal and model control, respectively. The model group exhibited activation of the AGEs/RAGE/HIF-1α axis with a significantly increased AGEs and RAGE expression compared with the Normal control. Additionally, rAAV-RAGE group further enhanced these targets expression and increased the FBG level, while RR-CO treatment downregulated these changes (Figures 6A–D). Meanwhile, compared with mice in the KK-Ay model control group, the key rate-limiting enzymes LDHC, PFK, and the transporter GLUT1 and GLUT3 related to glycolysis showed a more significant reduction in the rAAV-RAGE group. At the same time RR-CO could upregulate the expression of these critical proteins to some extent (Figures 6E–H).

## Discussion

Diabetes is a metabolic disease with a prevalence that has increased year on year (Ogurtsova et al., 2017). In addition to common complications such as diabetic nephropathy, retinopathy and neuropathy, more attention has and should be given to the damage by diabetes to the reproductive system (Khosravi et al., 2019). Male reproductive dysfunction will likely increase in the population over the coming years. One of the causes of this is metabolic disorders caused by unhealthy lifestyles, including excessive caloric intake. In metabolic disorders, DM has significant disruptive effects on the male reproductive system, including changes in testicular atrophy, sperm maturation disorders, sex hormone changes, etc. (Choubey et al., 2020). In the theory of TCM, the function of the kidney is closely related to reproduction. A deficiency of kidney-yin is core to pathogenesis of the DM-induced reproductive disorders. This study selected KK-Ay mice, a spontaneous diabetic nephropathy model of DM, in which there is glomerular hypertrophy and mesangial matrix deposition pathology. Studies have shown that KK-Ay mice, when induced by a high-fat diet, display pathological damage to the reproductive system of males, with morphological and functional damage to the genital system. HE staining of testis indicated a marked reduction in sperm cells, accompanied by disrupted seminiferous tubules for the DM model group. Moreover, the live sperm rate and testis/body weight ratio of KK-Ay mice decreased significantly compared with the control group. TUNEL staining also further revealed that the rate of apoptosis of spermatogenic cells in the DM model group was significantly higher than that of the normal control mice. All of these results indicated that the metabolic disorder caused by DM could indeed induce damage to the reproductive system, consistent with other previous reports (Rato et al., 2012; Rato et al., 2015b). However, the specific mechanisms responsible for these observations remain to be further elucidated.

Preliminary studies have shown that the leading causes of reproductive damage caused by DM are induction of inflammation and oxidative stress (Amaral et al., 2008; Khosravi et al., 2019; Chen Y. et al., 2020). We previously reported the deposition of AGEs in the testis, and that inflammation and oxidative stress were secondary to the activation of the AGEs-RAGE signaling pathway that plays an essential role in the reproductive malfunction induced by diabetes (Jiao et al., 2020). Some recent studies (Meneses et al., 2016; Yang et al., 2019) have also shown the vital role of metabolic factors that regulate normal spermatogenesis. Reduced glycolysis in the testis might lead to impaired spermatogenesis. Testicular metabolism is essential for sperm development and maturation.

In a normal physiological state, the testis is an organ with low oxygen. Spermatogenic cells, especially spermatocytes, mainly use lactate produced by anaerobic glycolysis (within SCs) as an energy substrate. SCs transport glucose from the tissue fluid of seminiferous tubules into cells through GLUT1 and GLUT3 glucose transporters. Glucose in SCs is metabolized under the action of HK, PFK1, pyruvate kinase (PK), etc. to produce pyruvate (via glycolysis) and then reduced to lactate under the act of LDH. The lactate is transported out of the cell by MCT4 of the SCs, then transported into the spermatogenic cells by MCT2 for intracellular metabolism and energy supply (Rato et al., 2012). Recent studies have shown that metabolic disorders caused by chronic hyperglycemia disrupted this close coordination of glucose metabolism between sperm cells and SCs, causing reproductive damage (Alves et al., 2015). Alves et al. observed that there was decreased expression of GLUT1, and PFK1 mRNAs in the testicular tissue of male patients with DM, accompanied by sperm damage (Alves et al., 2015). The lactate level and the activity of LDH were also significantly decreased. Rato et al. (Rato et al., 2015a) also reported that the damage of testicular sperm quality was accompanied by a significant decrease in lactate content and LDH activity in type 2 diabetic rats. The expression of PFK and LDH protein in SCs from type 2 diabetic mice was also significantly decreased.

In our study, LC-MS/MS was used to detect glycolysis metabolites. l-lactate, d-fructose 1,6-bisphosphate, ATP, glyceraldehyde 3-phosphate and pyruvate were decreased in the testicular tissue of the DM model, indicating that glycolysis was inhibited in the diabetic environment. Additionally, the expression of the glucose membrane transporters (GLUT1, GLUT3) and lactate transporter (MCT4) and activity of the glycolytic rate-limiting enzymes (HK, LDH and PFK) were evaluated within the testis. No significant differences were observed in testicular HK, LDH and PFK mRNA between the control and DM, but LDH activity within the testis was drastically reduced. Furthermore, significant protein reduction of GLUT1, GLUT3 and MCT4 was also observed in DM mice, results consistent with previous reports (Rato et al., 2015b). Overall, these studies suggested that glycolysis within testicular cells is inhibited in the diabetic environment, and therefore the energy substrate available for sperm cells decreased, and this is the basis of the deficiency of kidney yin that hindered the maturation of sperm cells.

The function of the kidney is closely related to reproduction in TCM. RR has the effects of clearing heat and cooling blood, nourishing yin and promoting fluid. CO can nourish the liver and kidney. The combined use of the two agents strengthens the function of tonifying the liver and kidney. Our previous studies focused on the role of the RR-CO combination in the prevention and treatment of diabetic nephropathy. RR-CO improved the pathological morphology of the kidney, and attenuated the damage to renal podocytes, endothelial cells and mesangial cells, through impact upon the AGEs-RAGE pathway in db/db diabetic male mice (Liu et al., 2015; Lv et al., 2016; Chen J. et al., 2020). In this study, we report that RR-CO improved the glomerular mesangial hypertrophy of KK-Ay diabetic mice. Pathological testicular damage as morphological changes and live sperm rates were also improved, which further validates the TCM theory of the “Kidney governing reproduction.” In addition, glycolysis within SCs produces lactate that is the energy substrate of sperm cells. The effect of RR-CO on this testicular glycolysis was also evaluated for the first time. We observed that RR-CO treatment could attenuate the decreased level of glucose metabolites such as l-lactate, d-fructose 1,6-bisphosphate, ATP, glyceraldehyde 3-phosphate and pyruvate that were reduced under the diabetic environment. Of further interest was that the reduced LDH activity and the GLUT1, GLUT3, MCT4 protein expression in the DM mice could also be partly recovered after RR-CO treatment. These data indicated that the herb pair RR-CO mitigated testicular damage by DM through enhancing testis glycolysis. However, the specific molecular regulatory mechanism remains to be further explored. We have previously reported that loganin-representative can affect components of CO and could abate DM-induced oxidative stress, inflammation and apoptosis in the testes through inhibiting AGEs-RAGE and the p38 mitogen-activated protein kinase (p38 MAPK) signaling pathway (Chen Y. et al., 2020). In this study, inhibitors of AGEs and RAGE, Ami and FPS-ZM1 were also used as a medicinal tool to investigate the regulation of the AGEs-RAGE axis on testicular glycolysis. The results showed that Ami and FPS-ZM1 improved the activity of LDH and increased the expression of MCT4 protein, while attenuating the testicular injury induced by diabetes. Moreover, the level of AGEs and protein RAGE expression was downregulated by Ami or FPS-ZM1 treatment. We constructed a model of RAGE overexpression KK-Ay mice, in which RAGE overexpression further inhibited the levels of glycolytic-related rate-limiting enzymes LDHC, PFK and the transporters GLUT1 and GLUT3 of testis. Still, FBG level was significantly increased in the RAGE overexpression group compared to the DM model group. Therefore, there is no linear correlation between systemically increased glucose concentrations and regional testicular metabolic changes. Liu (Liu et al., 2012) reported that the treatment of diabetic testicular lesions should control FBG level on the one hand, and improve testicular function on the other hand to reverse the pathological changes as much as possible. Accordingly, RR-CO treatment improved the level of testicular glycolysis to a certain extent. Consequently, we speculated that this promotion of testicular glycolysis by RR-CO in the diabetic environment might be related to the inhibition of the AGEs-RAGE axis. However, further in-depth studies are required to ascertain the critical molecular regulatory mechanisms.

Several studies have verified the effects of HIF-1α as a glycolytic promoter, one that promotes glycolysis by upregulating GLUT1, HK, LDH, and MCT4, etc. (Cerychova and Pavlinkova, 2018). In this study, it was found that while the expression of RAGE in the testis was significantly increased in KK-Ay mice, HIF-1α expression was significantly decreased. In addition, testicular glycolysis metabolites were also reduced. However, after treatment with RR-CO, RR, CO, or the inhibitor Ami, FPS-ZM1, HIF-1α expression was significantly increased, and the level of glycolysis in testicular cells was also promoted. Experimental evidence has suggested that AGE or its precursor MGO or the binding of AGE to its receptor RAGE, can contribute to HIF-1α repression in DM to different levels. MGO increases the degradation of HIF-1α via the carboxyl terminus of Hsp70 interacting protein. Also, MGO reduced HIF-1α deactivation by damaging its heterodimerization with HIF-1β (Ramalho et al., 2017; Choudhry and Harris, 2018). Taken together, all these preliminary findings indicate that HIF-1α is a crucial transcription factor in the regulation of testicular glycolysis when under a diabetic environment. AGEs can also downregulate the expression of HIF-1α after activation of RAGE.

In summary, the diabetic reproductive disturbance is closely related to kidney yin deficiency. Activation of AGEs-RAGE/HIF-1α-mediated the inhibition of glycolysis and subsequent lactic acid production, which could be the molecular basis of the kidney’s yin deficiency. Under the guidance of the theory of the “kidney governing reproduction,” the “nourishing Yin and tonifying kidney” herb pair RR-CO limited the levels of spermatogenic cell apoptosis, increased the live sperm rate, and attenuated testis pathological morphology caused by the diabetic environment through enhancing testicular glycolysis. The level of AGEs and protein RAGE expression was downregulated, and this was accompanied by increased expression of HIF-1α that collectively promoted glycolysis after treatment with RR-CO or via Ami or FPS-ZM1 treatment, suggesting the importance of the involvement of the AGEs-RAGE/HIF-1α axis in mediating the DM effects. Further investigations will be required to clarify the mechanism by which the AGEs-RAGE/HIF-1α axis can regulate glycolysis, and how RR-CO protects against this DM-induced testicular damage.

## Data Availability

The raw data supporting the conclusions of this article will be made available by the authors, without undue reservation.
