# Supplementary figures and images for: Combination of the Herbs Radix Rehmanniae and Cornus Officinalis Mitigated Testicular Damage From Diabetes Mellitus by Enhancing Glycolysis via the AGEs/RAGE/HIF-1α Axis
Source: Front Pharmacol. 2021 Jun 28;12:678300. doi: 10.3389/fphar.2021.678300 (PMC8273766; doi:10.3389/fphar.2021.678300)

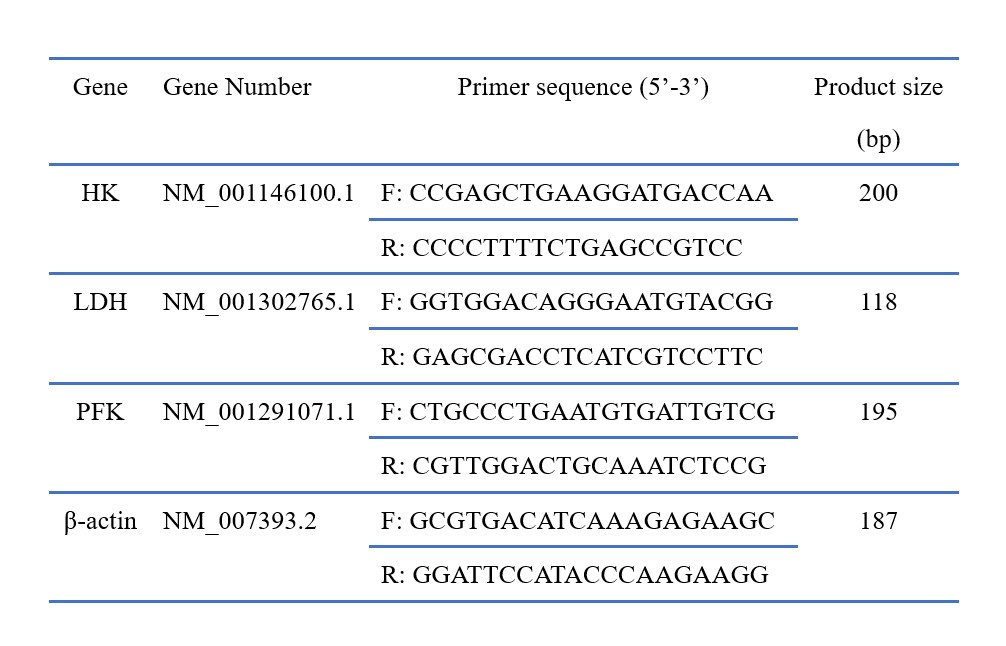

Supplement: Supplementary file 1 [file Image1.jpeg]
